# Waking Up to a New Model for Studying Neural Systems: What Emergence from Unconscious States Can Reveal about Brain Organization

**DOI:** 10.3389/fnsys.2017.00078

**Published:** 2017-10-17

**Authors:** Paul S. Garcia, Douglas L. Rothman, Susan M. Fitzpatrick

**Affiliations:** ^1^Department of Anesthesiology, Emory University, Atlanta, GA, United States; ^2^Anesthesiology and Research Divisions, Atlanta VA Medical Center, Atlanta, GA, United States; ^3^Department of Biomedical Engineering, Radiology and Biomedical Imaging, Yale University, New Haven, CT, United States; ^4^James S. McDonnell Foundation, St. Louis, MI, United States

**Keywords:** emergence, cognition, consciousness, anesthesia, sleep

Each day, anesthesiologists carry out an incredible feat when thousands of individuals are rendered *temporarily* unconscious by surgical anesthesia. Post-intervention, most of these individuals will “wake up” with their cognitive abilities, their memories, their knowledge of the world, and their sense of continuity of self, intact. The blank spot in the recovered person's conscious awareness is the period of time during which an individual is under anesthesia. What accounts for the ability of the central nervous system (CNS) to maintain a lifetime of information during a period of unconsciousness due to anesthesia when even brief episodes of unconsciousness due to injury or interruptions in cerebral metabolism can cause serious, irreversible losses of function? What makes it possible for the CNS to essentially “reboot” as the anesthetic is withdrawn? Does recovery of function following anesthesia reveal aspects of the organization of neural and cognitive systems? What factors could result in incomplete recovery of neural and cognitive systems and are there underlying clinical conditions that the brain more vulnerable to perturbations and thus make incomplete recovery more likely?

Surgical anesthesia provides an opportunity for studying changes in conscious states and a model for seeking the answers to the questions raised above. In a controlled clinical environment, anesthesiologists routinely pharmacologically manipulate a patient's brain activity from consciousness to unconsciousness and back. Within the clinical care setting it is not typical to consider such practices as “experiments” but there is data to be gathered from these situations that could be helpful in advancing our knowledge of brain organization. Anesthesia studies provide an alternative approach to the understanding of brain function that has shared interests with efforts to understand disruptions in emergence from unconsciousness in sleep disorders and coma.

Historically speaking, the cardiovascular and respiratory systems have more typically been the focus of anesthesia research than has been the functioning of the CNS—particularly with respect to higher-level, complex cognitive functions. As a result, significant advances have been made in airway techniques and physiologic monitoring, dramatically improving patient safety. Partly in response to these advances, attention is now shifting to the neural and cognitive effects of anesthetic administrations as evidenced by the heightened research interest in possible long-term consequences of commonly administered anesthesia drugs on the elderly and the developing brain. Progress on these issues will continue due to formation of: (1) the International Study of Post-Operative Cognitive Dysfunction (ISPOCD), a study group funded by the European Union (see JSMF, 2011) (2) the collaborative effort of the International Anesthesia Research Society (IARS), and (3) the US Food and Drug Administration SmartTots program (www.smarttots.org) dedicated to addressing scientific and clinical gaps regarding the safe use of anesthetics in children. Previous to these and other efforts, there were limited attempts for characterizing and monitoring awakening to cognitive and behavioral levels that are indistinguishable from a subject's baseline metrics. In our opinion, quantifying this sequence may have important clinical ramifications while revealing fundamental principles about brain organization.

Our aim is to facilitate research investigations focused on this topic especially those that involve neurophysiological measures with strong predictive potential for who will/not have full recovery after anesthesia as well as contribute to a more mechanistic understanding by which the brain recovers consciousness. “Neuro-anesthesia” is emerging as an important research direction tackling these questions of fundamental importance to neuroscience and cognitive science. A focused multi-disciplinary effort is now being brought to bear to answer questions raised in the context of emergence from anesthetic unconsciousness, including:

- In what ways, does anesthesia emergence resemble or depart from what is known about waking from coma or sleep?
- How does emergence from anesthesia reveal a predictable pattern reflective of anatomical, structural, and/or functional network organizations within the brain and CNS?
- What opportunities derive from cognitive and systems neuroscience approaches such as combining psychological testing with brain-sensing devices including electroencephalogram, infrared spectroscopy, or other imaging methods (e.g., fMRI, MRS, PET) that make it possible to address these research questions in humans?

A multi-disciplinary conversation on these and other questions has been the focus of several international workshops exploring what is and is not known about processes involved in regaining consciousness following surgical anesthesia, coma, and sleep (JSMF, 2011). Leading cognitive researchers, biophysicists, neurophysiologists, and clinicians explored common themes regarding clinical and research challenges surrounding emergence and recovery from anesthesia. It is our opinion, that the conclusions reached and future discovery areas are ripe for a concentrated and coordinated research investment. However, as of now, the major challenge of this field is that there are no clear criteria delineating clinical endpoints of waking up; nor are there standard tools with which to measure it.

## What does it mean to “wake up”? observations on regaining consciousness after anesthesia, coma, or sleep

Although the term “waking up” is used broadly to describe emergence from unconsciousness there is not broad consensus among researchers and clinicians of what it means to be awake. The three most familiar examples of transitioning from unconsciousness to consciousness are awakening from sleep, emerging from surgical anesthesia or recovery from traumatic or chemically-induced coma. Cross-condition data acquired as human subjects “wake up” or regain conscious awareness from each of these three states could expose shared neurobiological mechanisms or reveal crucial differences that provide insights into the neural systems required for conscious awareness and intact cognitive functioning. As opposed to sleep and the sleep stages (Dement and Kleitman, 1957), standard definitions of recovery from coma and emergence from anesthesia are not uniformly employed. Clinicians typically rely on subjective clinical judgment when they refer to “waking up” from these states. To advance scientifically and clinically requires that researchers and clinicians use a common lexicon, preferably based upon quantitative physical and cognitive measurements. Emergence from anesthesia is typically referred to as the transition from surgical anesthesia to a state with outward signs of cortical function (typically coordinated motor responses) while anesthesia recovery is the transition from the end of emergence to the clinician's subjective assessment of normal pre-operative neurocognitive function. In our experiences, emergence and recovery were not systematically presented nor were there universally adopted criteria by the workshop participants. In fact, it was often the case that individual research groups were primarily interested only in emergence and or recovery but not both. While research into the neurophysiologic signatures of anesthetic-induced unconsciousness (Lee et al., 2013; Akeju et al., 2014a,b; Pal et al., 2017) or the subsequent transition to waking (Solt et al., 2011; Safavynia et al., 2016) contributes to our understanding of the neural correlates of consciousness (Bonhomme et al., 2012; Pal et al., 2016), it is important to provide this work in the context of clinical anesthesiology and alongside studies focused on gaining a greater understanding of the potential adverse cognitive consequences (Mincer et al., 2017) or failure to reclaim the subject's pre-anesthesia baseline cognitive functions (Giattino et al., 2017). Standardized assessments for both emergence and recovery that go beyond simple cognitive and behavioral responses will be critical for understanding both processes.

## The future: opportunities, challenges, controversies, and recommendations

The integration of anesthesiology research with systems neuroscience research and the growing clinical specialization of neuroanesthesiology are increasingly offering important mechanistic and thematic insights. Still, a collaborative forum of leading cognitive researchers, biophysicists, neurophysiologists, anesthesiologists could only agree that “waking up” was a real and observable phenomenon lacking a well-characterized, quantifiable meaning across experimental and clinical settings. The field of neuroanesthesiology recognizes that waking up from different states (sleep, coma, anesthesia) share important mechanisms, however exploration of the similarities and differences about emergence across these different unconscious states is hampered by the lack of agreed upon definitions and end points (Rodriguez Moreno et al., 2010; Mota-Rolim and Araujo, 2013; Zahuranec et al., 2016) but as reflected in this special issue there is now dedicated efforts poised to make significant progress.

Through the work of several collaborative forums, discussions on the development of “standard” endpoints of anesthesia are being considered; including EEG signatures, psychomotor vigilance testing, functional imaging, and neurocognitive testing. The challenge, of course, is that many of the tests appropriate for research environments require specialized equipment or too much time to be practical in the clinical environment. As a start, we propose distinguishing the immediate waking from anesthesia (end emergence) as an appropriate response to an easily applied external stimuli. One example would be eye contact in response to as light tactile stimulation and/or auditory stimulation. The Observer's Assessment of Alertness/Sedation Scale (OAA/S) is one such scale validated for sedated patients (Chernik et al., 1990), but would need to be modified for some patients that are non-verbal after general anesthesia (Hernández-Gancedo et al., 2006). Defining when the actual complete period of anesthesia has ended, and the subject has returned to a pre-anesthesia baseline is more problematic, partly because a patient may be safe to discharge from the recovery room before *all* of the sedating effects of anesthesia have subsided and the complete recover trajectory is not documented. Quick cognitive screening assessments focused on memory or attention have been used to evaluate patients both pre-operatively (Culley et al., 2016, 2017) and in the recovery room (Card et al., 2015; Yang et al., 2017); but comparisons that follow the patients for longer periods of time are rarely made. More sophisticated testing involving neurocognitive tasks and brain imaging (e.g., fMRI) have been used mostly in the context of evaluating the potential for anesthesia drugs to exacerbate or lead to neurodegenerative disease (McDonagh et al., 2010; Goettel et al., 2016; Browndyke et al., 2017) and may not be appropriate for the operating room environment or patients not suspected of underlying impairments. We predict that assessments that require the re-establishment of sophisticated cortical networks (i.e., those requiring integration across multiple brain areas) would be convenient and sensitive for evaluating recovery as they have proven sensitive for neurodegenerative disease (Reilly et al., 2010). Additionally, theoretical data presented in this special issue suggest that specialized networks might possess little redundancy and are sensitive to low concentrations of anesthesia (Kim et al., 2017).

Most anesthesiologists recognize that simply rendering someone unconscious is trivial (e.g., alcohol, head trauma, injectable medication, etc.) however the magic of anesthesia is in safely waking patients up. To fully understand how waking occurs means tackling thorny philosophical and scientific questions. To what extent are aspects of consciousness graded or all-or-none? Are emergence circuits distributed or localized to a select few brain regions? To answer these questions will require anesthesia studies focused on correlating anatomically well-localized neurophysiologic measures with identifiable human behavior. Still, in the context of a person's recovery of their cognitive function and self-awareness the debates are fraught with practical implications. Anesthesia studies focused on correlating neurophysiologic measures with identifiable human behavior and neuroanatomical precision will be needed. The workshop discussions did generate general agreement that case studies and focused research on patients with more extreme phenotypes have been helpful in studying “waking up.” Prompting us to reconsider our model for clinical research—perhaps we should be embracing the complexity of human subjects in the many natural experiments carried out every day. One example might be a willingness to move beyond examining population statistics with a Gaussian perspective when much could be learned by analyzing on the data that falls outside of our expected ranges. In the well-studied case of sleep, both population and focused research on patients with extreme sleep phenotypes have clarified neural mechanisms supporting “waking up.” The study of outliers with clearly defined alterations in neuronal circuitry (for example due to genetic mutation) should provide similar insight into mechanisms of emergence and recovery from anesthesia.

There exists great potential and enthusiasm for growth in this interdisciplinary field of neuroanesthesia that exists at the intersection of cognitive neuroscience, anesthesiology, and consciousness. In a brief period, the field has already made contributions to standardizing measurements and reporting of emergence and recovery and identifying neural mechanisms that potentially differentiate normal from incomplete recovery of consciousness. However, much work remains to be done as it is abundantly clear that we know far more about using anesthesia to induce unconsciousness for surgery than about emergence and recovery from this state.

## Author contributions

All authors (PG, DR, SF) contributed equally to the intellectual content, writing of the manuscript, and the editing of the final version.

### Conflict of interest statement

The authors declare that the research was conducted in the absence of any commercial or financial relationships that could be construed as a potential conflict of interest.
